# Identifying Isl1 Genetic Lineage in the Developing Olfactory System and in GnRH-1 Neurons

**DOI:** 10.3389/fphys.2020.601923

**Published:** 2020-10-21

**Authors:** Ed Zandro M. Taroc, Raghu Ram Katreddi, Paolo E. Forni

**Affiliations:** Department of Biological Sciences, The RNA Institute, and the Center for Neuroscience Research, University at Albany, State University of New York, Albany, NY, United States

**Keywords:** olfactory neurons, vomeronasal sensory neurons, GnRH neurons, Islet-1/Isl1, Isl1 conditional knock-out, genetic lineage tracing, olfactory placode, neural crest

## Abstract

During embryonic development, symmetric ectodermal thickenings [olfactory placodes (OP)] give rise to several cell types that comprise the olfactory system, such as those that form the terminal nerve ganglion (TN), gonadotropin releasing hormone-1 neurons (GnRH-1ns), and other migratory neurons in rodents. Even though the genetic heterogeneity among these cell types is documented, unidentified cell populations arising from the OP remain. One candidate to identify placodal derived neurons in the developing nasal area is the transcription factor Isl1, which was recently identified in GnRH-3 neurons of the terminal nerve in fish, as well as expression in neurons of the nasal migratory mass (MM). Here, we analyzed the Isl1 genetic lineage in chemosensory neuronal populations in the nasal area and migratory GnRH-1ns in mice using *in situ* hybridization, immunolabeling a Tamoxifen inducible Isl1Cre^ERT^ and a constitutive Isl1^Cre^ knock-in mouse lines. In addition, we also performed conditional Isl1 ablation in developing GnRH neurons. We found Isl1 lineage across non-sensory cells of the respiratory epithelium and sustentacular cells of OE and VNO. We identified a population of transient embryonic Isl1 + neurons in the olfactory epithelium and sparse Isl1 + neurons in postnatal VNO. Isl1 is expressed in almost all GnRH neurons and in approximately half of the other neuron populations in the MM. However, Isl1 conditional ablation alone does not significantly compromise GnRH-1 neuronal migration or GnRH-1 expression, suggesting compensatory mechanisms. Further studies will elucidate the functional and mechanistic role of Isl1 in development of migratory endocrine neurons.

## Introduction

**FIGURE 1:**
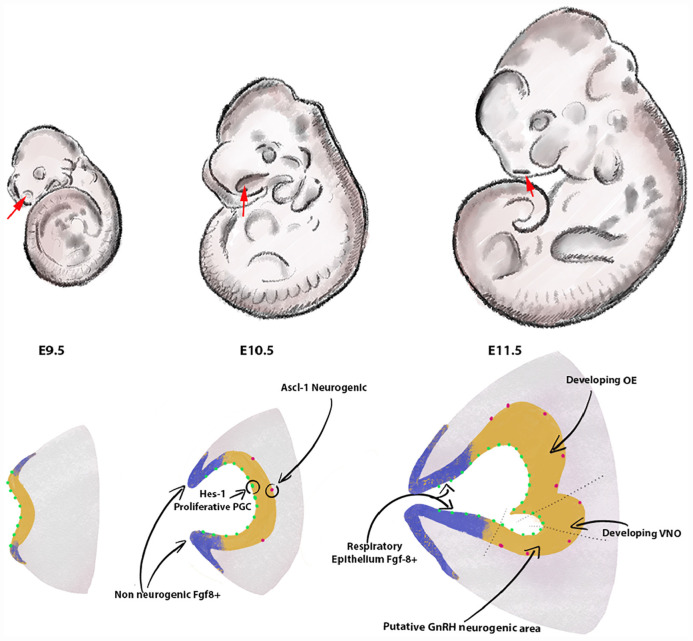
Formation of the olfactory pit. Drawings depict developing embryos between E9.5 and E11.5. The frontonasal areas where the olfactory placodes/pits form are indicated with red arrows. Cartoons in the lower row illustrate the olfactory placode ingression between E9.5 and E10.5 (chronal view) and a parasagittal view of the olfactory pit at E11.5. The lateral and rostral portions of the invaginating olfactory placode (blue) act as the main source of FGF8. These Fgf8 + areas progressively differentiate into respiratory epithelium. In the neurogenesis portion of the olfactory placode (yellow), proliferative Hes-1 + progenitors (green) give rise over time to neurogenic Ascl-1 neuronal progenitors. Arrows show the vomeronasal organ [olfactory epithelium (OE)] and putative neurogenic area of the GnRH-1ns at E11.5 in the developing OE. The size of the pit and cells is arbitrary and does not reflect natural proportions.

Cranial placodes are specialized regions of ectoderm, that give rise to the pituitary gland, sensory organs, and ganglia of the vertebrate head (Brugmann and Moody, 2005; Schlosser, 2006). They form as a result of specific expression patterns of transcription factors in pre-placodal ectoderm surrounding the anterior neural plate (Brugmann and Moody, 2005; Bailey and Streit, 2006). In mice, the olfactory placodes (OPs) are morphologically identifiable at embryonic day 9.5 (E9.5), as a bilateral ectoderm thickening in the antero-lateral region of the head (Figure 1). The transcription factors Oct1, Sox2, Pou2f1, Pax6, Eya1, Six1, Six4, Ngnr1,Hes1, Hes5, MASH1/Ascl1, Ngn1, and Gli3 have been identified to control various steps of OP induction/formation, neurogenesis, neuronal development, and expression of olfactory specific genes (Cau et al., 1997, 2002; Zou et al., 2004; Donner et al., 2007; Schlosser et al., 2008; Chen et al., 2009; Riddiford and Schlosser, 2016).

**FIGURE 2:**
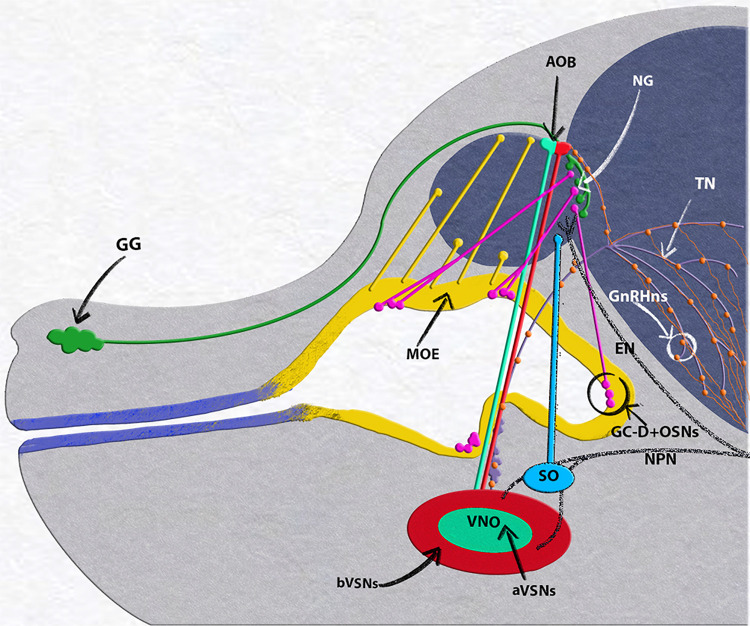
E15.5 mouse embryo, neuronal populations in the nasal area. Grueneberg ganglion (GG, green) and GC-D+/necklace (OSNs, magenta) project to the necklace glomeruli (NG). Olfactory neurons of the main olfactory epithelium (MOE, yellow) projecting to the main olfactory bulb (MOB). Vomeronasal organ (VNO), composed of basal (bVSNs, green) and apical (a-VSNs, red), projecting to anterior and posterior portions of the accessory olfactory bulb (AOB). Septal organ (SO, light blue) projecting to the ventral OB. Terminal nerve (TN, purple) projecting to the basal forebrain, GnRH-1ns, orange, scattered along the terminal nerve fibers from the vomeronasal area to the basal forebrain. Trigeminal nasopalatine (NPN) and ethmoidal nerve projections (EN) drawn in dark gray.

The olfactory pit of vertebrates generates neurogenic and non-neurogenic progenitors. The non-neurogenic portions of the olfactory pit give rise to the respiratory epithelium, which is located in the rostral olfactory pit, while the neurogenic portions give rise to specific neuronal and glial/support cells (Figures 1, 2). The respiratory epithelium, which is the main source of Fgf8, controls the development of the neural crest derived nasal mesenchyme and bones (Forni et al., 2013). Neurogenic waves in the olfactory pit of mice first gives rise to mostly migratory neurons (Fornaro et al., 2003; Forni et al., 2013) such as early pioneer olfactory neurons, neurons of the terminal nerve, including Gonadotropin releasing hormone-1 neurons (GnRH-1ns), NPY positive migratory neurons, and other neurons with unknown identity and function or neurons of the migratory mass (MM) (Schwanzel-Fukuda and Pfaff, 1989; Wray et al., 1989; Hilal et al., 1996; Fornaro et al., 2007). The nasal area of mice contains several other neuronal cell types that include specialized olfactory sensory neurons (OSNs) such as the guanylyl cyclase-D (GC-D) neurons of the necklace olfactory system (Luo, 2008; Mori et al., 2014; Greer et al., 2016), microvillar cells (MVCs) (Pfister et al., 2012), sensory neurons of the septal organ (SO) (Ma et al., 2003), the Grueneberg ganglion (GG) (Gruneberg, 1973; Schmid et al., 2010; Mamasuew et al., 2011; Matsuo et al., 2012; Moine et al., 2018), and cells forming the terminal nerve ganglion (TN) (Larsell, 1950; Brown, 1987; Jennes, 1987; Oelschlager et al., 1987; Schwanzel-Fukuda et al., 1987; Wirsig-Wiechmann, 2004; Taroc et al., 2017; Jin et al., 2019) including the GnRH-1ns (Schwanzel-Fukuda and Pfaff, 1989; Wray et al., 1989). The mechanisms and molecules that drive progenitors of the developing olfactory pit into early migratory cells types remain largely unknown.

Between E9.5 and E10.5, the OPs begin to ingress to form the olfactory pits that give rise to various cellular components of the nose during development (Cuschieri and Bannister, 1975b; Chen et al., 2009). The olfactory pit is largely populated by proliferative progenitors expressing the transcription factors Hes1 + /Hes5. These early progenitors undergo mitosis in the apical portion of the epithelium and give rise over time to neurogenic progenitors positive for the bHLH transcription factor Ascl-1 (Cuschieri and Bannister, 1975a,b; Cau et al., 2000; Taroc et al., 2020). Gli3 influences proliferative Hes1 + positive progenitors to give rise to Ascl-1 + neurogenic progenitors that eventually generate OSNs and vomeronasal sensory neurons (VSNs) (Cau et al., 2000; Taroc et al., 2020). Notably, Gli3 loss of function does not prevent neurogenesis of GnRH-1ns, suggesting that these have a distinct lineage from the olfactory neurons.

GnRH-1ns are the initial neuronal population to form in the developing olfactory pit (Wray et al., 1989; Fornaro et al., 2003). During embryonic development, GnRH-1ns migrate along the axons of the terminal nerve (Taroc et al., 2017) from the nasal area to the basal forebrain. Once in brain the GnRH-1ns control the release of gonadotropins from the pituitary gland (Forni and Wray, 2015). Aberrant development or function of GnRH-1ns causes hypogonadotropic hypogonadism (HH) (Cattanach et al., 1977), that can lead to a spectrum of reproductive disorders (Balasubramanian et al., 2010). GnRH-1 neuronal migration strictly depends on correct development and maturation of the neural crest derived olfactory ensheathing cells (Barraud et al., 2013; Pingault et al., 2013; Zhou et al., 2017; Wang et al., 2018). Cre genetic lineage tracing and BAC transgenic reporters have revealed extensive genetic heterogeneity among neurons that originate in the olfactory area of mice including the GnRH-1ns, suggesting a potential integration of neural crest cells in the developing OP (Forni et al., 2011b; Katoh et al., 2011; Suzuki and Osumi, 2015; Taroc et al., 2017; Shan et al., 2020). However, the mechanisms that give rise to the GnRH neurons and the required transcription factors for these regulatory processes remain unresolved.

The LIM-homeodomain transcription factor Isl1 has been recently identified in subsets of neuronal derivatives forming from the OP (Aguillon et al., 2018; Palaniappan et al., 2019; Shan et al., 2020). Isl1/2 expression occurs in GnRH expressing neurons and other putative neurons of the terminal nerve (Aguillon et al., 2018; Palaniappan et al., 2019; Lund et al., 2020; Shan et al., 2020) in chick, zebrafish, mouse, and humans. In zebrafish (Aguillon et al., 2018), Isl1/2 is expressed in terminal nerve GnRH-3 neurons associated with the olfactory epithelium. These neuro-modulatory cells, which are non-migratory in fish, exert the same physiological function as migratory GnRH-1ns in mice (Forni and Wray, 2015). The terminal nerve and MM in birds contains heterogeneous populations of (1) cells that express GnRH-1 but are negative for Isl1/2 and Lhx2, (2) cells only expressing Lhx2, (3) cells only positive for Isl1, and (4) cells that co-express Lhx2, Isl1/2, GnRH-1 (Palaniappan et al., 2019). In birds, cells from the TN are also positive for Isl1 and Lhx2 that differ from the GnRH-1ns. A third study in mouse (Shan et al., 2020) also confirmed Isl1/2 immuno-reactivity on GnRH-1ns at early stages of GnRH neuronal development (E11.5). A subset of GnRH-1ns, positive for Wnt1Cre genetic lineage (Forni et al., 2011b), were negative for Isl1 immunoreactivity.

One prediction is that Wnt1Cre tracing, a controversial neural crest marker, and Isl1 immunoreactivity define two distinct embryonic lineages for GnRH-1ns. The rival hypothesis is that the Wnt1Cre positive subpopulation of GnRH-1ns differ in its timing and/or expression levels of Isl1 from the majority of GnRH-1ns (Forni et al., 2011b; Shan et al., 2020). Understanding the molecular cascade that establishes the genetic lineage of different cell types is a fundamental step to identify potential cellular differences between cell types and to unravel the molecular mechanisms underlying neurodevelopmental pathologies. Here, we examined which regions in the OP express Isl1/2 and analyzed Isl1 genetic lineage in different neuronal types in the nasal area of mice. We further generated Isl1 conditional KO mice to study the role of Isl1 in GnRH development.

## Materials and Methods

### Animals

Isl1^flox^(Isl1tm2Sev/J) (Sun et al., 2008), Isl1^CreERT^(Isl1tm1(cre/Esr1^∗^)Krc/SevJ) (Laugwitz et al., 2005), Isl1Cre (Isl1tm1(cre)Sev/J) (Yang et al., 2006), Rosa26tdTomato (B6.Cg-Gt(ROSA)26Sortm14(CAG-tdTomato)Hze/J) (Madisen et al., 2010), and GnRH-Cre (Gnrh1-cre 1Dlc/J) (Yoon et al., 2005) mouse lines were all purchased from JAX. Genotyping was conducted following the suggested primers and protocols from JAX. Mice of either sex were used for ISH and IHC experiments. All experiments involving mice were approved by the University at Albany Institutional Animal Care and Use Committee (IACUC).

### Tamoxifen Treatment

Tamoxifen (Sigma–Aldrich), CAS # 10540−29−1, was dissolved in Corn Oil. Tamoxifen was administered once via intraperitoneal injection at indicated developmental stages. Tamoxifen was administered at 180 mg/kg based on the weight of the pregnant dam on the day of injection.

### Tissue Preparation

Embryos were collected from time-mated dams where the emergence of the copulation plug was taken as E0.5. Collected embryos were immersion-fixed in 3.7% formaldehyde/PBS at 4°C for 3–5 h. Postnatal animals were perfused with 3.7% formaldehyde/PBS. All samples were then cryoprotected in 30% sucrose overnight, then frozen in O.C.T (Tissue-TeK), and stored at −80°C. Samples were cryosectioned using CM3050S Leica cryostat and collected on Superfrost plus slides (VWR) at 14 μm thickness for embryo’s and 16 μm for post-natal tissue.

### Immunohistochemistry

Primary antibodies and dilutions used in this study were: rabbit- α-Isl1(1:1000, Abcam), mouse-α-Isl1/2 (1:100, DSHB), SW rabbit-α-GnRH-1 (1:6000, Susan Wray, NIH), goat-α olfactory marker protein (OMP; 1:4000, WAKO), rabbit-α-phospho-Histone-H3 (1:400, Cell Signaling), goat-α-Sox2 (1:400, R & D systems), rat-α-phospho-Histone-H3 (1:500, Abcam), mouse-α-Ki67 (1:500, Cell Signaling), rabbit-α-DsRed (1:1000, Clontech), goat-DsRed (1:1000, Rockland), HuC/D 8 μg/ml (Molecular Probes). Antigen retrieval was performed in a citrate buffer prior to incubation with rabbit-α-Isl1, mouse-α-Isl1/2, rabbit-α-phospho-Histone-H3, rat-α-phospho-Histone-H3, mouse-α-Ki67. For immunoperoxidase staining procedures, slides were processed using standard protocols (Forni et al., 2013) and staining was visualized (Vectastain ABC Kit, Vector) using diaminobenzidine (DAB) in a glucose solution containing glucose oxidase to generate hydrogen peroxide; sections were counterstained with methyl green. For immunofluorescence, species-appropriate secondary antibodies were conjugated with Alexa-488, Alexa-594, or Alexa-568 (Molecular Probes and Jackson Laboratories) as specified in the legends. Sections were counterstained with 4’,6’-diamidino-2-phenylindole (1:3000; Sigma–Aldrich) and coverslips were mounted with Fluoro Gel (Electron Microscopy Services). Confocal microscopy pictures were taken on a Zeiss LSM 710 microscope. Epifluorescence pictures were taken on a Leica DM4000 B LED fluorescence microscope equipped with a Leica DFC310 FX camera. Images were further analyzed using FIJ/ImageJ software. To remove auto-fluorescent blood cells in Figure 9, FIJ/ImageJ function image calculator was used. Each staining was replicated on at least three different animals for each genotype.

### *In situ* Hybridization

Digoxigenin-labeled cRNA probes were prepared by *in vitro* transcription (DIG RNA labeling kit; Roche Diagnostics) from the following templates: Isl1 from Allen Brain Atlas (Probe RP_080807_04_G12). *In situ* hybridization was performed as described (Lin et al., 2018) and visualized by immunostaining with an alkaline phosphatase conjugated anti-DIG (1:500), and NBT/BCIP developer solution (Roche Diagnostics).

### Cell Quantifications

GnRH-1/Isl1 double IHC quantifications were performed at E11.5 and E15.5 parasagittal non-serial sections on a single slide using the Rabbit-α-GnRH-1 primary and Mouse-α-Isl1/2 primary antibodies. Embryonic co-localized cell counts on Isl1Cre or Isl1Cre^ERT^ lineage traced animals were done on E14.5 and E15.5, respectively, parasagittal non-serial whole head sections within the nasal region on a single slide where we see co-localization of both markers indicated (DsRed/tdTomato GnRH-1, or HuCD). Post-natal OMP cell counts were done on P8 coronal nose sections. To get the density of mature OSNs positive for constitutive Isl1 lineage tracing at P8, number of OMP positive neurons colocalized for tdTom were counted in the caudal olfactory epithelium and calculated the area of the OMP positive cells within the traced epithelium. In VNO, to get the percentage of mature VSNs positive for constitutive Isl1 lineage tracing, first OMP positive cells colocalized for tdTom were counted. To get the total OMP positive cells in VNO section, density of OMP cells in smaller areas of VNO was determined and extended to the total area of OMP positive cells in the VNO.

GnRH-1 cell counts were performed on GnRH-1Cre/Islet1^flox/flox^, Isl1^CreERT/flox^, and control at E15.5 on two immunostained non-serial slides.

### Validation of Conditional Isl1 Knockout

The Isl1^flox^ animals have the lox-p sites flanking exon 4 of the Isl1 gene which codes for the homeodomain and consists of the amino acids in positions 181–240. To detect conditional loss of Isl1^flox^ in GnRHCre/Isl1^flox/flox^ mutants, we performed immunolabeling using the mouse anti-Isl1/2 (DSHB) primary antibody. This antibody recognizes the amino acids 178–349 of the Isl1 protein and was previously shown to be unable to detect Isl1^flox^ (Isl1tm2Sev/J) mice, after Cre mediated recombination (Sun et al., 2008). However, in Isl1^CreERT^ [Isl1tm1(cre/Esr1^∗^)Krc/SevJ] which is null mouse model, the amino acids in positions 181–240 are maintained, therefore this null protein is still detectable in Isl1 null cells. Since Isl1^CreERT^ (Laugwitz et al., 2005) and Isl1^flox^ alleles (Sun et al., 2008) have been targeted in different gene regions, we could not validate recombination efficiency of Isl1^CreERT^/^flox^ cKOs.

### Statistical Analyses

All statistical analyses were carried out using GraphPad Prism7 software. All cell counts were averaged ± standard error (SE) among animals of the same age and genotype. Means ± SEs were calculated on at least three animals per genotype. The statistical difference between genotypes and groups was determined using an unpaired student’s *t*-test.

## Results

### Isl1 Expression in Proliferative Progenitors and Neurons During Early Development of the Olfactory Pit

**FIGURE 3:**
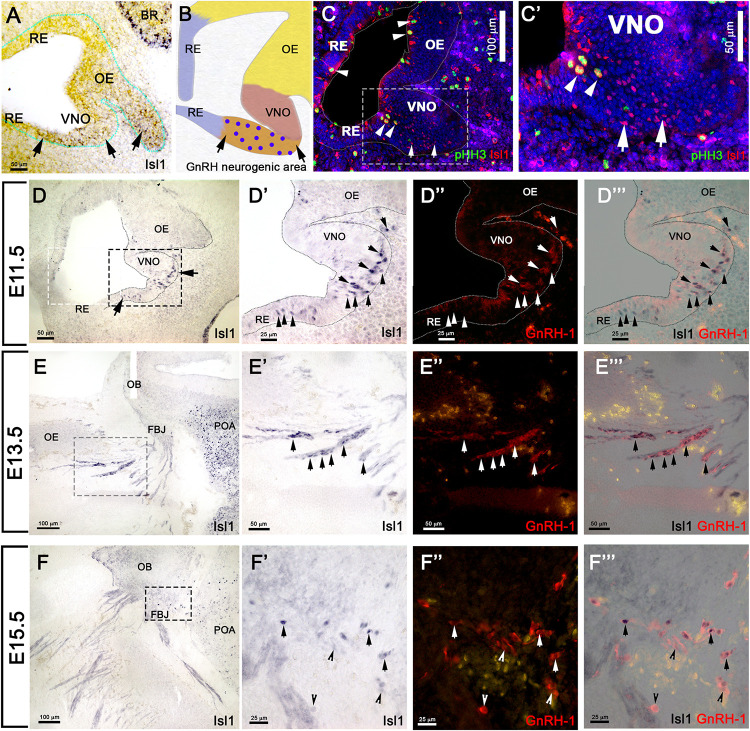
Isl1 expression in the OP is restricted to GnRH neurons and a few other cells types. **(A)** E11.5 *in situ* hybridization anti-Isl1 mRNA shows Isl1 expression in the ventral portion of the developing olfactory pit proximal to the VNO. Arrows indicate putative GnRH neurogenic area. Only spare cells positive for Ilset1 could be detected in the OE (arrowhead). **(B)** Cartoon illustrating the putative areas giving rise to RE, OE, VNO, and the putative GnRH neurogenic area. Blue dots indicate GnRH-1ns. **(C,C’)** Immunostaining anti-Isl1 (red) and the mitotic marker pHH3 (green) shows Ilset1 expression in sparse proliferative cells in the apical portion of the OP (arrowheads) and in cells in the putative GnRH neurogenic area ventral to the VNO (boxed) compared to **(A,B)**. **(D)** Isl1 immunoreactivity in the putative GnRH neurogenic area (arrows, compared to **(A,B,C)**. **(D’–D”’)** Isl1 expression (black) in newly formed GnRH neurons(arrows) and in GnRH-1 negative cells (arrowheads). Isl1 expression (black) in migrating GnRH-1ns in the (arrows) in the nasal area at E13.5 **(E–E”’)** and in the brain at E15.5 **(F–F”’)**. OE, olfactory epithelium; OB, olfactory bulb; FBJ, forebrain junction; POA, preoptic area; RE, respiratory epithelium; VNO, vomeronasal organ; BR, brain.

Using *in situ* hybridization at E11.5, we found Isl1 mRNA expression in the more caudal region of the putative developing olfactory epithelium and proximal to the developing VNO (Figure 3A). We previously described the latter as the region where GnRH-1ns likely form and become immunoreactive (Figure 3B) (Forni et al., 2013). By pairing immunohistochemistry against Isl1/2 and the mitotic marker phospho-histone-3 (PHH3) at E11.5, we found (Figures 3C,C’) some proliferative progenitors in the apical regions of the developing pit that were also positive for Isl1. Isl1/2 protein expression was consistent with the mRNA expression pattern (Figures 3A,C). However, strong Isl1/2 immunoreactivity appeared localized in sparse nuclei ventral to the developing VNO and the putative respiratory epithelium (Figures 3C,D). Immunolabeling with anti Isl1/2 and GnRH-1 at E11.5, E13.5, and E15.5 (Figures 3D–F”’) indicated a dynamic Isl1/2 expression across GnRH-1ns (Shan et al., 2020). In fact, we could identify at E11.5 approximately 89% (SE ± 3.33%) of GnRH-1 + cells positive for Isl1/2 immunoreactivity (Figures 3D–D”).

### Differential Lineage Among Olfactory, Vomeronasal, and GnRH-1 Neurons

**FIGURE 4:**
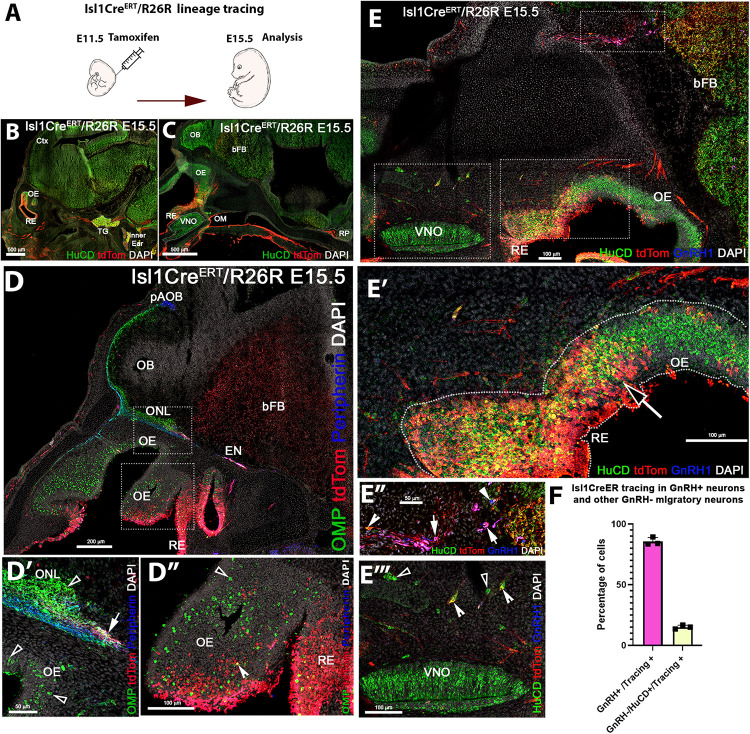
Temporally controlled Isl1 lineage tracing at E11.5 primarily highlights cells of the respiratory epithelium and GnRH-1ns. **(A)** Cartoon illustrating experiential strategy for Ilset-1CreERT/R26RTdTomato lineage tracing. Tamoxifen induction of Cre recombiantion at E11.5, analysis at E.15.5. **(B,C)** HuC/D/DsRed double immunostaining shows recombination (tdTomato) in the trigeminal ganglion (TG), the inner ear as well as in cells of the Rathke’s pouch (RP). **(D–D”)** In the developing nose recombination appears to be mostly restricted to the respiratory epithelium (RE) and ethmoidal (EN) trigeminal projections. **(D–D”)** Anti tdTomato; OMP; Peripherin immunostaining showed that OMP positive olfactory projections of the main olfactory bulb. Peripherin positive projections to the accessory olfactory bulb appear negative for td Tomato expression. However, tdTomato positive fibers (**D’** white arrow), likely belonging to the trigeminal/ethmoid nerve (see EN in **D**), appeared to project to the ventral OB. **(D”)** OMP and tdTomato immunoreactivity showed nearly complete absence of OSNs positive for Isl1 tracing. Neurons in the olfactory area appeared mostly negative for Isl1 lineage **D”**, sparse OMP + tdTomato + cells (Notched arrowhead) could be detected at the border between olfactory and respiratory epithelium, boxed area see **D’**. Pan neuronal marker HuC/D shows neurons of the developing vomeronasal organ (VNO), mostly negative for Isl1 lineage **(E’)**. HuC/D + neurons at the border between OE and RE **(E’)** migratory neurons, GnRH-1ns **(E”)** and HuC/D + only **(E”’)** were positive for Isl1 tracing. **(F)** Quantification of recombination in migratory GnRH-1 + and HuC/D GnRH-1 negative neurons. OM, oral mucosa; RP, Rathke’s Pouch; Ctx, cortex; OB, olfactory bulb; bFB, basal Fore Brain; pAOB, putative Accessory Olfactory Bulb.

As we observed sparse and a heterogeneous distribution of Isl1 mRNA and protein expression in the developing olfactory pit at E11.5 (Figure 3) and found Isl1/2 protein expression with the proliferative cell marker PHH3, we decided to investigate which cells formed from the Isl1/2 + proliferative cells. We utilized a tamoxifen inducible Isl1Cre^ERT^ knock-in mouse line (Laugwitz et al., 2005) mated with a sensitive Rosa-reporter (Madisen et al., 2010). Isl1Cre^ERT^ allows temporal control and restricts Cre mediated recombination to the time of Tamoxifen injection. Pregnant dams were treated with a single injection of Tamoxifen (180 mg/kg) at E11.5 and embryos were analyzed 4 days later (Figure 4A). At E15.5, we observed Isl1 recombination in trigeminal nerve, inner ear, Rathke’s pouch, and oral mucosa (Figures 4B,C) (Hutchinson and Eisen, 2006; Sjodal and Gunhaga, 2008; Tanaka et al., 2011; Huang et al., 2013). We also found Isl1 tracing in the developing nasal area (Figures 4D–E”) in sparse OMP + sensory neurons of both the main olfactory and vomeronasal epithelium. Notably, Isl1Cre + neurons positive for the pan neuronal marker HuC/D were found at the border between the respiratory epithelium and sensory epithelium (Figures 4E,E’). We found extensive Cre recombination penetrance throughout the nasal respiratory epithelium (Figures 4D,D’).

We detected putative nasopalatine and ethmoidal trigeminal axons positive for Isl1 throughout the nasal area and tangential to the olfactory bulb (Figures 4B–D’). By analyzing the olfactory projections via OMP and Peripherin immunostaining in combination with anti tdTomato staining, we confirmed that Isl1Cre recombination occurred in neurons that appeared to bundle with the olfactory neurons. Notably, most Isl1Cre^ERT^ traced axons did not appear to project to the main or accessory olfactory bulb (Figures 4D,D’). Analysis of the olfactory epithelium showed a very sparse colocalization between OMP and Isl1Cre mediated recombination. However, immunostaining against the pan neuronal marker HuC/D showed the presence of Isl1 + neurons within the developing olfactory epithelia (Figures 4E,E’). Triple immunostaining against GnRH-1, tdTomato, and the pan neuronal protein HuC/D showed that temporally controlled Isl1Cre^ERT^ recombination at E11.5 yielded 85% (SE ± 1.88%) of GnRH-1ns, while only 15%(SE ± 0.91) of the migratory (HuC/D + /GnRH−) cells were Isl1 positive. These data suggest that Isl1 expression at E11.5 (Figures 3A–C) is mostly limited to progenitors or precursors of GnRH-1ns and the subsets of other neurons of the MM.

### Analysis of Constitutive Isl1 Cre Genetic Lineage Tracing at E14.5

**FIGURE 5:**
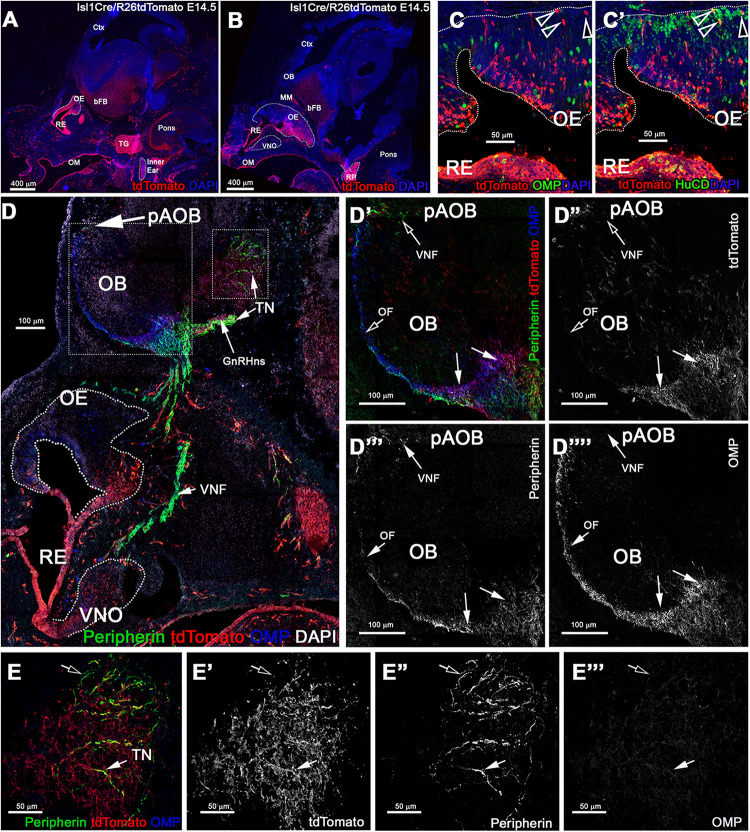
Constitutive Isl1 lineage tracing highlights: sparse neurons the OE, VNO, neuronal projections to the ventral OB, GnRH neurons, and some TN fibers. **(A,B)** Immunostaining against tdTomato of Isl1Cre/R26tdTomato lineage traced embryos at E14.5, showing recombination the trigeminal ganglion (TG), inner ear, Rathke’s pouch (RP), the oral mucosa (OM), respiratory epithelium (RE), and in sparse cells in the OE and VNO. **(C)** Anti-tdTomato; OMP and **(C’)** anti-tdTomato; HuC/D staining shows that though almost all the OMP + cells were negative for tracing (arrowheads) **(C)**, those were neurons positive for HuC/D (arrowhead). **(D–E”’)** Immunostaining against tdTomato, OMP, and Peripherin to highlight the projections of the developing olfactory system reveal that Isl1 tracing **(D’–D””)** is mostly absent in the OMP + olfactory fibers (OF) and Peripherin + vomeronasal fibers (VNF) innervating the olfactory bulb (OB). GnRH-1 and other cells of the migratory mass positive for the tracing were detected invading the brain along the terminal nerve (TN). **(E–E”’)** Peripherin positive TN fibers in the brain. Some fibers appear positive for the tracing (solid arrow) while other Peripherin fibers appear to be negative (empty arrow). MM, migratory mass; bFB, basal Fore Brain; OE, Olfactory Epithelium; VNO, Vomeronasal Organ.

Tamoxifen controlled Cre recombination at E11.5 indicated that few neuronal progenitors and sparse neurons in the OE and VNO were positive for Isl1 as the olfactory pit forms, while the respiratory epithelium, oral mucosa, and the majority of the GnRH-1ns resulted expressing Isl1. To further perform a temporally unbiassed lineage tracing for Isl1, we exploited a traditional Isl1Cre knock-in mouse line. In these mice recombination is expected to occur whenever Isl1 is expressed (Yang et al., 2006). We performed a Cre/Rosa reporter lineage analysis Isl1^Cre±^/R26^tdTomato±^ at E14.5 and P8. After temporally controlled recombination (Figure 4), constitutively active Isl1 Cre at E14.5 (Figure 5) showed recombination in the trigeminal nerve, cells of the inner ear (Radde-Gallwitz et al., 2004), the Rathke’s pouch forming the anterior pituitary gland (Castinetti et al., 2015), the oral mucosa, and the nasal respiratory epithelium.

During gross visual observation, we noticed that more cells positive for Isl1 lineage occurred in both olfactory and vomeronasal epithelia compared to that after temporally controlled Cre activation (compared Figures 4, 5).

### Isl1 Cre Recombination in GnRH and Terminal Nerve Neurons

**FIGURE 6:**
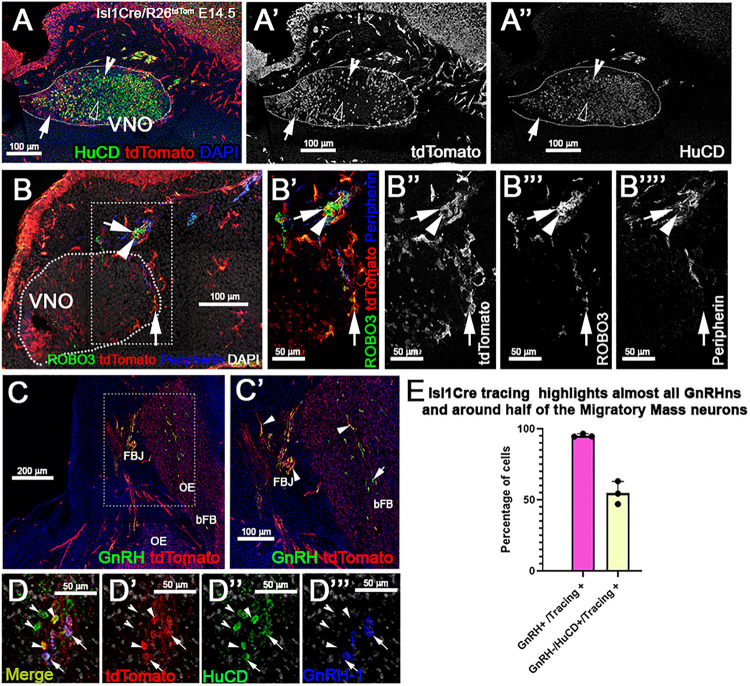
Isl1 constitutive lineage tracing highlights GnRH-1ns, some cell bodies of the TN, and other migratory neurons. **(A–A”)** HuC/D and tdTomato immunostaining of Isl1Cre^±^/R26tdTomato VNO at E14.5 show some neurons within the VNO positive (empty arrowhead) and negative (notched arrowhead) for Isl1 recombination. Population of tdTomato + non-neuronal cells positive for tracing (arrow). **(B–B””)** Anti-Robo3; tdTomato and Peripherin labeling shows that a portion of putative terminal nerve cell bodies located within and proximal to the VNO are highlighted by Isl1 recombination (arrow) while a portion do not (arrowhead). **(C–C’)** GnRH and tdTomato staining shows recombination in the majority of the GnRH-1ns (arrowheads). **(D–D””)** Recombination in ∼ half of the (GnRH−;HuC/D +) migratory neurons (arrows). **(E)** Quantification of Isl1Cre recombination in migratory GnRH + and HuC/D GnRH-1 negative neurons. VNO, vomeronasal organ; OE, olfactory epithelium; FBJ, fore brain junction; bFB, basal fore brain.

At E14.5 we performed OMP, Peripherin, and tdTomato immunostaining. We observed labeled olfactory and terminal axonal projections (Figures 5D–E”’). In the olfactory system, we noticed that Isl1 Cre tracing could be seen in fibers forming bundles with the olfactory neurons after temporally controlled Isl1 Cre recombination (Figures 5D’–D”’). However, most axons positive for recombination at this stage appeared to project only to the ventral portions of the olfactory bulb with some verging on invading the basal forebrain (Figures 5E–E”’). The terminal nerve enters the brain ventral to the olfactory bulb (Taroc et al., 2017). Fibers of the putative terminal nerve associated with Isl1 + migratory GnRH-1ns. Using immunostaining against peripherin and tdTomato, we visualized Isl1 + positive cell somas and fibers of GnRH-1ns migrating on peripherin positive axons of the TN. Some putative TN’s axons showed sparse positivity for tracing (Figures 5E–E””). To confirm Isl1 Cre tracing in cells of the terminal nerve, we immunostained against the antigen Robo3, which is selectively expressed by these cells (Taroc et al., 2017). Robo3/tdTomato staining confirmed the presence of cell bodies from putative TN cells, proximal and within the developing vomeronasal organ, some of these appeared to be positive for Isl1 lineage. We also observed TN fibers, positive for Robo3, in the brain (data not shown). GnRH-1, tdTomato, and HuC/D (Figures 6D–D”’) immunostaining confirmed Isl1 lineage in virtually all GnRH-1ns (95%) (SE ± 0.68), while 55% (SE ± 4.63) of the HuC/D migratory neurons that were negative for GnRH-1, were actually positive for Islet1 tracing (Figure 6E).

### Postnatal Analyses of Isl1Cre Lineage in the Nasal Cavity

**FIGURE 7:**
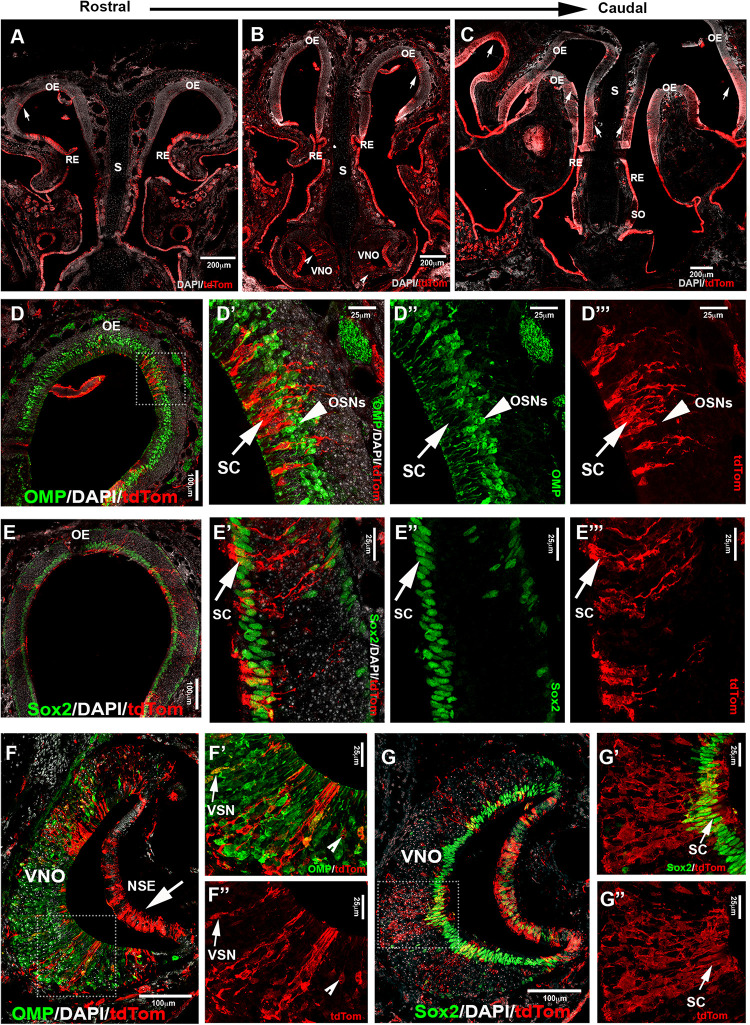
Lineage tracing of Isl1 positive cells postnatally highlights neuronal and non-neuronal cells in OE and VNE. **(A–C)** Immunofluorescence against tdTom showing Isl1 tracing in olfactory epithelium (OE) from rostral toward the caudal ethmoid turbinates. Arrows **(A,B,C)** show tracing in OE. **(B)** Notched arrowheads show tracing in VNO. **(D–D”’)** Double immunofluorescence against OMP (green) and tdTom (red) shows no colocalization in neurons of the OE. **(E–E”’)** Double immunofluorescence against Sox2 (green) and tdTom (red) shows Isl1Cre tracing (arrow marks) in the sustentacular cells of the OE. Sox2 is present in both globose basal cells that are present in the base of the OE and sustentacular cells in the upper layers of the OE. **(F)** Double immunofluorescence against OMP (green) and tdTom (red) shows Isl1 tracing in both non-sensory epithelium (NSE—arrow mark) and sensory epithelium of the VNO. **(F’,F”)** Arrow marks show colocalized mature vomeronasal neurons (VSNs) and notched arrow heads show traced neurons that are not labeled with OMP. **(G–G”)** Double immunofluorescence against Sox2 (green) and tdTom (red) shows Isl1 tracing in sustentacular cells in the VNO. S, septum; SO, septal organ; SC, sustentacular cells; OSNs, olfactory sensory neurons.

During embryonic development, we observed Isl1 recombination in neurons in both olfactory and vomeronasal epithelia. So, we analyzed the pattern of constitutive Isl1 Cre recombination in postnatal animals. We performed double immunostaining against tdTomato and OMP at postnatal day 8 and noticed that most olfactory epithelium neurons did not express OMP and Isl1. However, we did find sparse neurons positive for Isl1 tracing in the most caudal ethmoid turbinates [0.12 (±0.03 SEM) cells/1000 μm^2^] (Figures 7D–D”’). Isl1Cre positive sustentacular cells were found close to the border between the respiratory epithelium and sensory olfactory epithelium in the ventral–lateral zones of the OE and along the nasal septum (S) in all the sections from rostral to caudal. At visual gross observation, we noticed more pronounced Isl1 + lineage in sustentacular cells in the caudal OE along the lateral ethmoid turbinates (Figures 7A–C). These data imply spatial/regional heterogeneity in gene expression among sustentacular cells (Brann et al., 2020).

**FIGURE 8:**
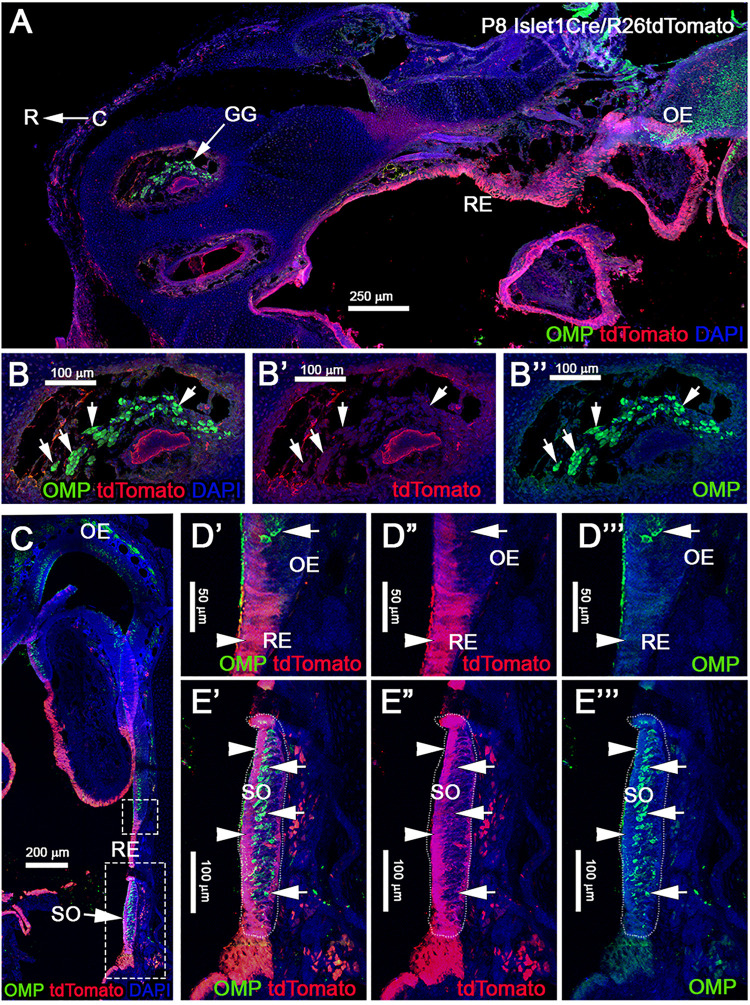
Post-natal Isl1 constitutive lineage tracing is not expressed in other nasal sensory neurons. **(A)** Isl1Cre/R26tdTomato P8 parasagittal section of the nose immunostained against OMP and tdTomato shows that Isl1 recombination is limited to the respiratory epithelium even in post-natal stages. Neuronal sensory populations, such as the Gruenberg Ganglion (GG) **(B–B”)**, is negative for recombination. **(C)** Coronal view of Isl1Cre/R26tdTomato at P8, stained against OMP and tdTomato shows Isl1 tracing restricted to the non-sensory cells and cells of the respiratory epithelium. **(D–D”’)** Magnification from **C** of the border between olfactory and respiratory epithelium shows Isl1Cre tracing restricted to the respiratory epithelium (arrowhead), while OMP + neurons are negative (arrow). **(E–E”’)** Septal organ (SO) from **C** indicates Isl1 recombination is restricted to the non-sensory (arrowheads) cells, while OMP + neurons are negative (arrows). R, rostral; C, caudal; RE, respiratory epithelium; OE, olfactory epithelium.

Analysis of postnatal vomeronasal epithelia showed a different scenario from that in the OE. Immunostaining against TdTom indicated Isl1 tracing in both non-sensory epithelium (NSE) and sensory epithelium of the VNO. In the sensory epithelium, staining against OMP and tdTom showed that sparse recombination in 3.74% (±1.06 SEM) of the neurons (Figures 7F–F”). The vomeronasal neurons positive for tracing were variously distributed in apical and basal regions. Sox2 and tdTomato immunostaining revealed, as observed in the OE (Figures 7E–E”’), that Islet1 tracing was detectable in sustentacular cells (Figures 7G–G”). Since constitutive Isl1 lineage showed cells positive in olfactory and vomeronasal epithelia, we further investigated if Isl1 recombination also occurred in neurons of the SO and Gruenberg ganglion (GG). By performing immunostaining anti-OMP in postnatal animals, we found that both neuronal populations were negative for lineage tracing (Figure 8). Immunostaining with anti-CART and -PDE2A antibodies also revealed an absence of Isl1 expression in Necklace glomeruli cells (NGCs) (data not shown).

**FIGURE 9:**
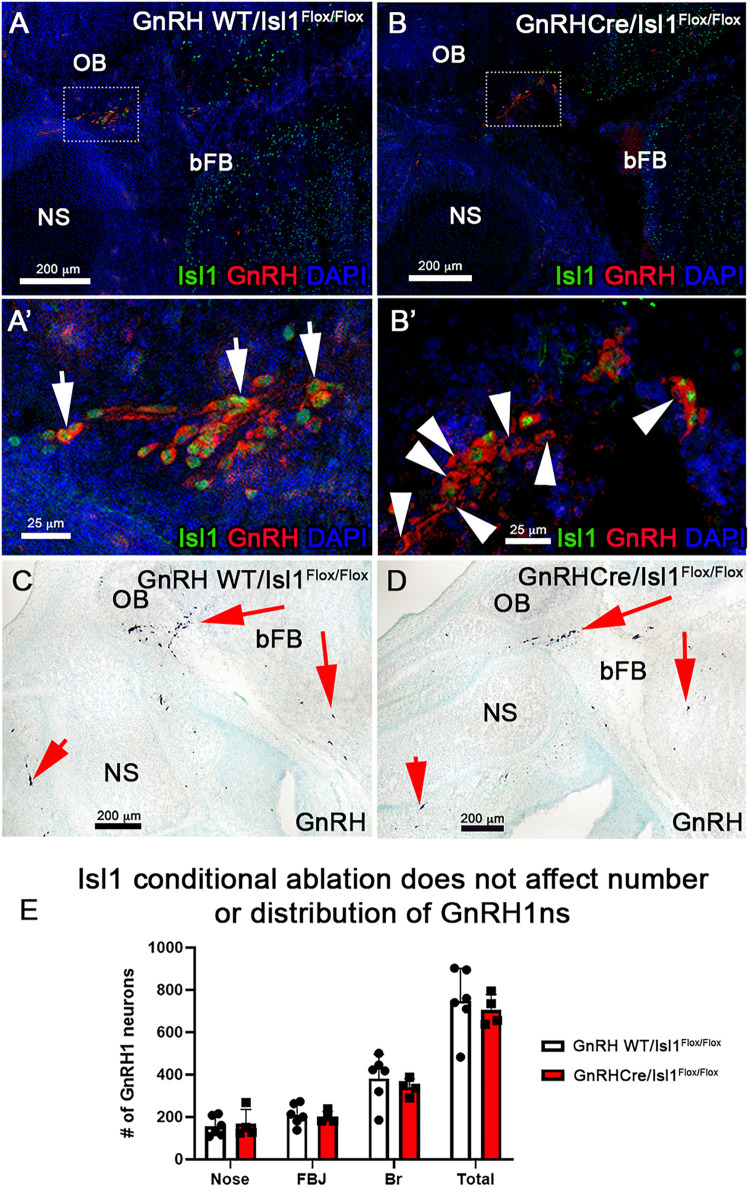
Conditional loss of Isl1 in GnRH-1ns has no effect in their migration or development. Isl1/2 and GnRH double IF on E15.5 WT **(A–A’)**, where all GnRH-1ns express Isl1/2 (arrows) and cKO **(B–B’)** sections show that some GnRH-1ns lose the expression of Isl1 (arrowheads). **(C,D)** Immunohistochemistry of GnRH-1 on WT and cKO sections shows comparable distribution of cells positive for GnRH-1ns. **(E)** Quantification of cKO shows no changes in the number or distribution of GnRH-1ns. NS, nasal septum; OB, olfactory bulb; bFB, basal forebrain.

### Conditional Isl1 Loss of Function in GnRH-1ns

Isl1 is important for differentiation, cell migration, survival, and axonal targeting of neurons of the peripheral nervous system (Thor et al., 1991; Thaler et al., 2004; Sun et al., 2008). Our data revealed strong Isl1/2 expression in developing migrating GnRH-1ns (Figures 3, 4, 6). To test if Isl1 plays a role in GnRH-1ns migration or controls GnRH expression, we generated GnRH-1Cre/Isl^flox/flox^ conditional mutants. Embryos were analyzed at E15.5, which is the stage at which the majority of the GnRH-1ns have already migrated in the brain (Forni et al., 2011a). Double immunostaining against Isl1/2 and GnRH confirmed detectable Isl1 ablation in 38% (SE ± 5.28%) of GnRH-1ns (Figures 9A–B’), while Isl1 in controls was detectable in virtually all migratory GnRH-1ns. In GnRH-1Cre/Isl^flox/flox^ conditional mutants, GnRH-1ns negative for Isl1 immunoreactivity were found in the nasal area, forebrain junction, and the brain. These data suggest that Isl1 expression is not required for GnRH-1 peptide expression nor GnRH-1 neuronal migration. Quantification of GnRH-1ns distribution in nasal area, forebrain junction, and brain indicated an overall distribution of GnRH-1ns comparable with the one of controls (Figure 9E).

## Discussion

Cellular derivatives of the OPs play central roles in respiration, chemosensory detection, sexual development, fertility, and inter- and intra-species social behaviors. Understanding the basic mechanisms that define cellular diversity and underly normal and pathological development of the OP derivatives is of high clinical importance. Isl1 expression can occur in various neurogenic placodes (Begbie et al., 2002; Zhuang et al., 2013); however, a comprehensive analysis of Isl1 expression remains unknown. Isl1, which is important for differentiation, cell migration, survival, and axonal targeting of neurons in the peripheral nervous system (Thor et al., 1991; Thaler et al., 2004; Sun et al., 2008), has a very high upregulation during GnRH-1 neuronal differentiation (Lund et al., 2020). So, Isl1 remained a potential suitable genetic marker to label derivatives of the OP (Shan et al., 2020). Here, we sought to trace Isl1 expression and lineage in the developing nasal area using *in situ* hybridization, immunohistochemistry (Figure 3), temporally controlled Is1CreRT recombination (Figure 4), and constitutive Isl1Cre^ERT^ genetic lineage tracing (Figures 5–8).

### GnRH-1 Lineage Tracing in the Developing Nasal Area

Using *in situ* hybridization and immunohistochemistry, we detected Isl1 expression at E11.5 in the putative GnRH neurogenic area, ventral to the developing VNO (Figures 3A–D), in newly formed GnRH-1ns and the developing olfactory epithelium (Figures 3D–F”’). At E11.5, Isl1 immunoreactivity occurred in 89%(±3.33%) of GnRH cells, in line with published reports (Shan et al., 2020). However, histochemistry at E13.5 and E15.5 showed strong Isl1/2 immunoreactivity in virtually all GnRH-1ns, suggesting that Isl1 expression is not limited to specific subpopulations of GnRH cells (Shan et al., 2020). These data suggest a dynamic expression of Isl1 with more GnRH-1 cells positive across development.

At E11.5 we also found the existence of proliferative cells positive for Isl1 in the developing olfactory pit. Tamoxifen controlled Isl1Cre^E*RT*^ tracing at E11.5 showed Isl1 lineage in putative GnRH progenitors/newly formed GnRH-1ns, cells of the respiratory epithelium, few proliferative progenitors in the developing olfactory pit, neurons at the border between respiratory epithelium and developing olfactory epithelium and sparse neuronal (HuC/D+) cells in the OE and VNO. Notably in this experiment we analyzed Isl1Cre^E*RT*^/R26R recombination 4 days after Tam treatment. This means that, when we observed the histological samples, we identified either cells that expressed Isl1 at the moment of Tam injection or cells that derived from progenitors expressing Isl1 at time of Tam injection.

In line with what observed after temporally Tam controlled recombination at E11.5, constitutively active Isl1Cre recombination showed that, during embryonic development (Figures 5, 6), Isl1 tracing highlights neurons in OE proximal to the border with RE, neurons in the vomeronasal organ, cells of the RE, GnRH-1ns, migratory neurons negative for GnRH, and sustentacular cells in olfactory and vomeronasal organ.

These data indicate that Isl1 expression/lineage is limited to specific subsets of olfactory placodal derivatives. In contrast to prior observations during embryonic development, Isl1Cre recombination analysis of postnatal Isl1Cre/R26R mice showed only sparse neurons positive for Isl1 tracing in the most caudal regions of the olfactory sensory epithelium, while cells positive for Isl1 tracing were still found in the VNO, respiratory epithelium, and in sustentacular cells of both OE and VNO (Figure 7). No Isl1 expression or lineage was in GC neurons and GG (Figure 8). These data suggest that Isl1 positive neurons in the OE at the border with the RE during embryonic development (Figures 4, 5) either migrated out of the epithelium as a part of the MM or died, even though we could still detect Isl1 expression/lineage in some vomeronasal organ neurons (Figures 7F–F”). So, we further defined the fate of the Isl1 neurons detectable in the OE during embryonic development. During embryonic development, we could also detect axonal projections positive for Isl1 traversing through the nasal area (Figures 4, 5). Some axonal projections likely belonged to the nasopalatine projections of the trigeminal nerve (Figures 3–5). Although both Isl1^CreE*RT*^ and Isl1Cre induced recombination in Peripherin + fibers projected to the ventral OB, the majority of OMP + fibers projecting to the MOB and AOB appeared negative for Isl1 tracing (Figures 4, 5). We did not pursue a detailed analysis of olfactory projections in postnatal animals, due to technical challenges in tracing Isl1 recombination in mitral cells of the MOB.

### Isl1 in the GnRH-1 System

During embryonic development, GnRH-1ns migrate from the developing olfactory pit into the hypothalamus. Once in the preoptic area, GnRH neurons (GnRH-1ns) control the hypothalamic–pituitary–gonadal hormonal axis and reproductive development. Defective GnRH-1 release is the cause of HH, a condition that negatively impacts normal body development, social interactions, the ability to procreate, and physical performances (Burmeister et al., 2005; Yin and Gore, 2006; Maruska and Fernald, 2011; Zhang et al., 2013). HH associated with congenital anosmia (impairment of the sense of smell) is clinically defined as Kallmann syndrome (Kallmann, 1944; Legouis et al., 1991; Guioli et al., 1992; Hardelin et al., 1992; Incerti et al., 1992; Dode et al., 2003; Pitteloud et al., 2007). Though postnatal development of the olfactory system has been extensively studied, we still ignore many aspects regarding early development of the olfactory pit derivatives.

Previous studies in chick and mice indicate that soon after the OPs form they give rise to migratory olfactory pioneer neurons, and to several other neuronal populations including the terminal nerve cells and the GnRH-1ns. Placodal derived migratory neurons and the neural crest derived olfactory ensheathing cells form the MM (Barraud et al., 2013, 2010).

We previously showed that the fibers of the terminal nerve, upon which the GnRH-1ns migrate, are distinct from the OSN and VSN (Taroc et al., 2017, 2019, 2020). What progenitors give rise to the GnRH neurons, and what transcription factors control their onset are still open questions. Moreover, if what we define as GnRH neurons is one homogenous neuronal population is also a matter of debate.

Lineage tracing at different developmental stages suggests strong and consistent Isl1 expression in GnRH-1ns and cells of the MM (Figures 3, 4, 6). While the terminal nerve that projects from the vomeronasal area to the basal forebrain appeared to have heterogeneous Isl1 expression/genetic lineage tracing (Figure 6), Isl1 has been reported to be important in neuronal differentiation, cell migration, survival, and axonal targeting of various neuronal types (Thor et al., 1991; Thaler et al., 2004; Sun et al., 2008). The consistent Isl1/2 expression in developing and migratory GnRH-1ns (Figures 3, 4, 6), which is conserved from fish to humans prompted us to test its developmental role.

We first made an attempt to conditionally KO Isl1, which as a whole KO is embryonic lethal, by mating Isl1^CreE*RT*^ mice (Figure 4), which are Isl1^*null*^ heterozygous, with Isl1^flox^. After Tamoxifen injection at E11.5, we analyzed Isl1^CreE*RT*/flox^ mice and controls at E15.5 (as in Figure 4). After Tamoxifen treatment, Isl1^CreE*RT*^ extensively recombines in the developing GnRH-1ns (Figure 4). Surprisingly Tam + Isl1^CreE*RT*^/Isl1^flox^ embryos did not show any obvious phenotype related to GnRH-1ns (Supplementary Material). However, for this model, we could not verify the efficiency of recombination because antibodies unable to detect the floxed Isl1 truncated protein were still recognizing the Isl1^CreE*RT*^ protein product (see section “Materials and Methods”). For this reason, we report these as supplementary observations. In order to further test the role of Isl1 in GnRH-1 neuronal development, we generated GnRH-1Cre/Isl1^flox/flox^ conditional mutants. Here, we could document complete loss of Isl1 immunoreactvity in around ∼40% of GnRH-1ns (Figure 9). Notably most of the anti Isl1 Abs have some level of cross reactivity with Isl2, therefore we performed *in situ* hybridization against exon 4 of Isl1 paired with anti GnRH-1 immunofluorescent staining (not shown). These experiments suggest similar penetrance of recombination as indicated by IF. Notably, also in GnRH-1Cre/Isl1^flox/flox^ mutants, we did not observe obvious defects in GnRH-1 neuronal migration nor in GnRH-1 expression (Figure 9). In fact, GnRH-1ns negative for Isl1 were found in both nasal area and brain. Total number and overall distribution of GnRH-1 was comparable to controls. These data suggest that Isl1 alone is not necessary for GnRH-1 expression or neuronal migration. We believe that our conditional Isl1KO might partially compensate Isl1 loss-of-function through Isl2 expression. Notably, Isl1 and Isl2 have been shown to be required for the expression of neuropilin-1 (Nrp1) (Lee et al., 2015). Moreover, it was recently demonstrated that selective loss of Nrp1 in GnRH-1ns leads to a total increase in the number of GnRH-1ns in mouse (Vanacker et al., 2020). In line with these observations in our Isl1^CreERT/Flox^ cKO embryos, we observed a trend with an increase in the total number of immunoreactive GnRH-1ns (Supplementary Figure S1). Since loss of Nrp1 expression is more dramatic in Isl1/Isl2 double knockouts (Lee et al., 2015), we predict that Isl1/Isl2 double knockouts could have increased number of GnRH-1ns as reported for Nrp1 null mice (Vanacker et al., 2020).

## Conclusion

Our data indicate very selective expression of Isl1 in neurons forming in the OP including the GnRH-1ns, various migratory neuronal populations and some cells in the VNO, terminal nerve and sustentacular cells on the more medial and lateral portions of the OE. Isl-1 immunoreactivity and genetic tracing suggest that Isl-1 expression, though dynamic, is common feature for virtually all GnRH-1ns (Shan et al., 2020) and several other neurons of the MM. If all these early migratory neurons derive from similar pre-placodal subregions should be further investigated. Performing GnRH-1ns specific Isl1 ablation in GnRH-1ns we did not detect obvious cell specification, differentiation, survival, or migratory defects (Shan et al., 2020) at embryonic development stages suggesting a dispensable role for Isl1 in GnRH neurons.

Our results leave open the question of what is the physiological role of this transcription factor in murine GnRH-1ns development and function. Further studies focused on Isl1 role in postnatal developmental and fertility can give definitive insights.

## Data Availability Statement

All datasets presented in this study are included in the article/Supplementary Material.

## Ethics Statement

The animal study was reviewed and approved by the Institutional Animal Care and Use Committee (IACUC).

## Author Contributions

ET set the mouse mating, collected samples, prepared histological samples, performed immunostaining and imaging, and analyzed data at embryonic developmental stages. RK prepared histological samples and performed immunostaining and analyses of postnatal animals. ET and PF designed the experiments, wrote the article, and prepared figure panels. All authors contributed to the article and approved the submitted version.

## Conflict of Interest

The authors declare that the research was conducted in the absence of any commercial or financial relationships that could be construed as a potential conflict of interest.

## Supplementary Material

Supplementary Figure 1 Isl1Cre^ERT^/Isl1^Flx^ conditional knockout at E11.5 does not affect GnRH1ns. GnRH IHC on WT **(A)** and cKO **(B)** sections show that between each genotype the distribution of GnRH-1ns looks similar. **(C)** Quantification of the number of GnRH-1ns in whole embryo heads shows no significant regional changes or in total cell number.
