# Supplementary figures and images for: Identifying Isl1 Genetic Lineage in the Developing Olfactory System and in GnRH-1 Neurons
Source: Front Physiol. 2020 Oct 21;11:601923. doi: 10.3389/fphys.2020.601923 (PMC7609815; doi:10.3389/fphys.2020.601923)

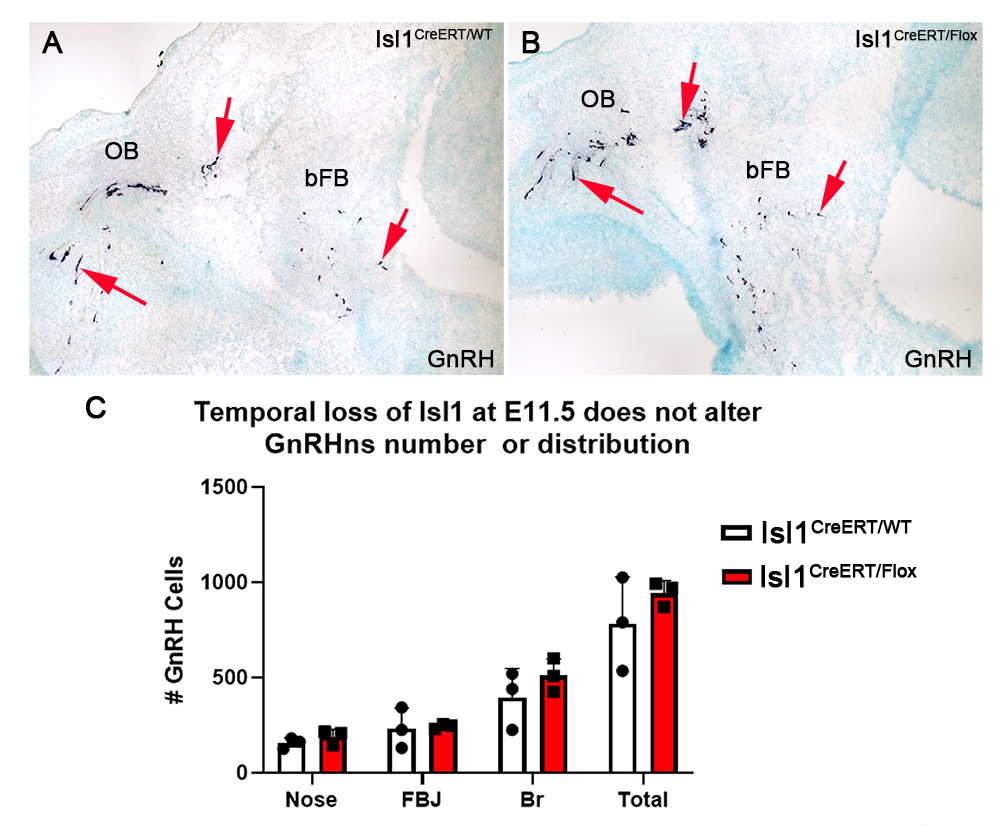

Supplement: Supplementary Figure 1 — Isl1CreERT/Isl1Flx conditional knockout at E11.5 does not affect GnRH1ns. GnRH IHC on WT (A) and cKO (B) sections show that between each genotype the distribution of GnRH-1ns looks similar. (C) Quantification of the number of GnRH-1ns in whole embryo heads shows no significant regional changes or in total cell number. [file Image_1.TIF]
